# Effect of an audiovisual message for tetanus booster vaccination broadcast in the waiting room

**DOI:** 10.1186/1471-2296-12-104

**Published:** 2011-09-28

**Authors:** Caroline Eubelen, Fannette Brendel, Jean-Luc Belche, Anne Freyens, Sophie Vanbelle, Didier Giet

**Affiliations:** 1 Centre de Santé Intégrée des Carrières, rue Vieille Voie de Liège, 1, B-4140 Sprimont, Belgium; 2 Department of General Medicine, University of Liège, CHU Sart-Tilman, B-4000 Liège, Belgium; 3 Department of General Medicine, University of Toulouse, Faculté de Médecine de Toulouse Rangueil, Route de Narbonne, F-31062 Toulouse, France; 4 Department of Methodology and Statistics, University of Maastricht, Peter Debeyeplein 1, 6229 HA Maastricht, The Netherlands

## Abstract

**Background:**

General practitioners (GPs) often lack time and resources to invest in health education; audiovisual messages broadcast in the waiting room may be a useful educational tool. This work was designed to assess the effect of a message inviting patients to ask for a tetanus booster vaccination.

**Methods:**

A quasi experimental study was conducted in a Belgian medical practice consisting of 6 GPs and 4 waiting rooms (total: 20,000 contacts/year). A tetanus booster vaccination audiovisual message was continuously broadcast for 6 months in 2 randomly selected waiting rooms (intervention group - 3 GPs) while the other 2 waiting rooms remained unequipped (control group - 3 GPs). At the end of the 6-month period, the number of vaccine adult-doses delivered by local pharmacies in response to GPs' prescriptions was recorded. As a reference, the same data were also collected retrospectively for the general practice during the same 6-month period of the previous year.

**Results:**

During the 6-month reference period where no audiovisual message was broadcast in the 4 waiting rooms, the number of prescriptions presented for tetanus vaccines was respectively 52 (0.44%) in the intervention group and 33 (0.38%) in the control group (p = 0.50). By contrast, during the 6-month study period, the number of prescriptions differed between the two groups (p < 0.0001), rising significantly to 91 (0.79%) in the intervention group (p = 0.0005) while remaining constant in the control group (0.38% vs 0.39%; p = 0.90).

**Conclusions:**

Broadcasting an audiovisual health education message in the GPs' waiting room was associated with a significant increase in the number of adult tetanus booster vaccination prescriptions delivered by local pharmacies.

## Background

GPs and family doctors are responsible for providing health information to their patients [1]; however, in many practices, no strategy exists to effectively perform this service. Lack of time is often mentioned to explain the absence of the service as well as lack of training [2-4]. Given the growing number of elderly patients consulting GPs and of patients being treated for chronic diseases in primary care settings [5], there is a need among primary care providers to look into innovative ways to inform patients more efficiently about health promotion and education.

Patients spend varying amounts of time in the physicians' waiting rooms; thus, waiting rooms offer an opportunity to expose patients to health information. Several studies have already assessed the impact of health education strategies located in the waiting room [6-10]. The current study purposed to investigate the effect of an audiovisual message repeatedly broadcast in the GPs' waiting rooms. Specifically, it focussed on the impact of a short video sequence about tetanus booster vaccinations on subsequent requests for these vaccinations.

Tetanus vaccination coverage in Belgium remains unsatisfactory: 64.2% of the patients attending an emergency room have protective immunity [11]. This is consistent with the results of the 2001 Federal Ministry Health National Inquiry where only 68% of the adult participants claimed to be fully vaccinated [12]. Another study showed that, in the Belgian countryside, the proportion of subjects aged > 11 years with complete tetanus vaccination was estimated at 51.8% [13]. In the Belgian healthcare system, where patients can freely choose (and change) their physician, the initiative to evoke a tetanus booster vaccination, except in the case of an infected wound or bite, is mainly left to the patient. This prompted us to initiate an audiovisual strategy to foster among patients attending a GP visit a demand to revise their current tetanus immunization coverage.

## Methods

The study was conducted in a general medical practice consisting of six GPs located in the same building of a little rural town of about 13,000 inhabitants in Belgium. All GPs were working in the fee-for-service Belgian healthcare system. The annual number of patient contacts per GP ranged between 3,000 and 4,500 (adults and children included). The patient population of each GP was considered homogeneous and the six GPs were not aware of any socio-demographic differences between them. The practice building had six consulting rooms, of which three were associated with a block of two waiting rooms and the other three with another block of two waiting rooms; each practitioner used the same consulting room and hence associated waiting rooms.

The study was designed to assess the effect of an audiovisual message inviting patients to ask for a tetanus booster vaccination. In that purpose, one block of two waiting rooms was randomly selected (coin toss) and equipped with an audiovisual device for a 6-month period (July - December 2008), while the other block of two waiting rooms was not. The six GPs were not blinded for the randomisation. The audio-visual device, consisting of a flat screen television and loudspeakers connected to a central computer, displayed in a continuous and repeated manner a variety of health education messages (e.g., home accident prevention advices, counselling for physical activities). One of the messages broadcast by the audiovisual device was an invitation to consider a tetanus booster vaccination. After a short presentation about tetanus condition, prophylaxis and benefits of vaccination, patients were encouraged to ask their doctor to check their tetanus vaccination status during the consultation. The "intervention" group included all patients consulting the 3 GPs who used the two equipped waiting rooms. The "control" group consisted of all patients visiting 3 GPs who used the two other unequipped waiting rooms. With the help of the five pharmacists working in the municipality, all adult tetanus vaccine prescriptions from the six GPs that were collected by all five local pharmacists during the study period were recorded (adult tetanus vaccines in Belgium are bivalent or trivalent vaccines for tetanus and diphtheria and/or pertussis). Only one vaccine dose per patient was recorded, regardless of the number of doses required as part of remedial tetanus vaccination schemes.

At the end of the study, the same specific data, namely the number of patients attending each of the six GPs as well as the number of adult tetanus vaccine prescriptions from the six GPs, were collected retrospectively for the corresponding 6-month period of the previous year (July - December 2007). This period, in which the four waiting rooms operated without any audiovisual equipment, served as a reference of the general practice for the study.

Statistical methods: The number of contacts was recorded for each GP during each 6-month period. The proportion (%) of prescriptions was calculated for each study group (intervention and control) and each 6-month period (July-December 2007 and July-December 2008). Proportions were compared by the classical chi-square test. Results were considered significant at the 5% critical level (p < 0.05).

Ethical approval was obtained from the "Commission Ethique du Département de Médecine Générale de Midi Pyrénées", Toulouse, France.

## Results

The number of contacts, the number and percentage of prescriptions recorded for each physician are displayed in Table 1 according to study group (intervention and control) and 6-month time period (2007 and 2008).

**Table 1:** Number of patient contacts and number and percentage of doses prescribed recorded for each physician according to waiting rooms (intervention and control) during the 6-month reference period (no audiovisual equipment) and the 6-month study period (audiovisual equipment in the intervention group and none in the control group).

Waiting rooms	Reference period (July-December 2007)				Study period (July-December 2008)		
		**Number of contacts**	**Number of doses**	**Percent**	**Number of contacts**	**Number of doses**	**Percent**
Intervention group	GP1	3382	9	0.27	3154	31	0.98
	GP2	3936	30	0.76	3931	37	0.94
	GP3	4533	13	0.29	4381	23	0.52

	Total	11851	52	0.44^(a)(b)^	11466	91	0.79^(b)(d)^

Control group	GP4	2816	19	0.67	2630	14	0.59
	GP5	2413	5	0.21	2438	6	0.25
	GP6	3495	9	0.26	3575	14	0.39

	Total	8724	33	0.38^(a)(c)^	8643	34	0.39^(c)(d)^


Proportions were compared two by two by the chi-squared test and corresponding p-values are indicated by the same superscript as footnotes.

^(a) ^0.44 vs 0.38 (p = 0.50)

^(b) ^0.44 vs 0.79 (p = 0.0005)

^(c) ^0.38 vs 0.39 (p = 0.90)

^(d) ^0.79 vs 0.39 (p < 0.0001)

During the July - December 2007 reference period (no audiovisual device installed), a total of 20,575 contacts was reported for the general medical practice. When classifying these contacts according to the study groups, there were 11,851 contacts for the three GPs in the intervention group and 8,724 contacts in the control group. The corresponding numbers of prescriptions recorded were 52 (0.44%) and 33 (0.38%) in the intervention and control groups, respectively. No significant difference was found between these percentages (p = 0.50), hence emphasizing the homogeneity of the two groups with respect to tetanus vaccine prescription during the reference period. For the entire practice, the July - December 2007 percentage of prescriptions amounted 0.41% (95%CI: 0.32% - 0.50%).

During the actual study period carried out between July and December 2008 (audiovisual message in one group and none in the other), 11,466 contacts were recorded in the intervention group and 8,643 in the control group, yielding a total of 20,109 contacts for the general medical practice. In the intervention group, the number of prescriptions rose to 91 (0.79%) as compared to 52 (0.44%) during the reference period. This corresponds to a highly significant increase (p = 0.0005). By contrast, in the control group, the number of prescriptions amounted 34 (0.39%), a value close to 33 (0.38%) observed in the reference period (p = 0.90). Overall, the proportions of prescriptions differed markedly between the intervention and the control groups (0.79% versus 0.39%; p < 0.0001).

## Discussion

Our results indicate that exposure to an audiovisual message about tetanus booster vaccination in a waiting room was associated with an increase in the number of prescriptions presented for tetanus vaccinations. We believe that the audiovisual message broadcast in the waiting rooms attracted the patients' attention and encouraged them to take the active step of asking for the vaccination

Our results contradict those of a study conducted in a similar setting in Canada in 1995 [14], which focused on posters in waiting rooms encouraging patients to request tetanus vaccinations. The authors did not find that the posters had a significant impact.

The use of audiovisual devices in waiting rooms has been the subject of several studies, in which positive effects were found in non-general practice waiting rooms [15-17]. Furthermore, the effects in general practices of audiovisual devices broadcasting information on subjects other than vaccination have also been studied [18,19]. Some authors concluded that audiovisual messages had a positive impact, in particular when reinforced by an additional intervention [17,20,21]. Our study showed that exposure to a stand-alone audiovisual message about tetanus booster vaccination can be associated with an increase of number of prescriptions for tetanus vaccination.

Clearly the present study suffers from a number of shortcomings. Although no detailed socio-demographic and clinical characteristics of the patient population were available, the six GPs were unaware of major reasons for the two groups to differ. An accurate list of patients cared for by a medical practice and the socio-demographic characteristics of the served population are not readily known because of the following reasons. Healthcare delivery in Belgium is mainly based on the principles of independent medical practice, i.e. independent medical practitioners are paid via fee-for-service payment and there is free choice of doctor by the patient. Patients may register with a doctor of their choice, but registration is not compulsory. Therefore, this situation forced us to use as numerator the number of annual contacts, children and adults included. The fact that the participating GPs were not blinded may be an additional source of bias but almost impossible to avoid in the present circumstances. The investigators relied on their professional and personal behaviour.

It is unclear that the audiovisual message led to a change of patients' behaviour regarding tetanus booster vaccination request. We used an indirect means of measuring change, because we recorded the number of GPs' prescriptions presented to pharmacies by patients in both groups, rather than the number of patient requests. These prescriptions may be subject to various influences, such as the GPs' motivation. Furthermore, providing patients with booster vaccines in a pharmacy does not necessarily mean that the vaccine was injected; the patient may forget to bring the prescribed vaccine dose to their next GP's visit, and it may never be injected.

We also found that, although the audiovisual message was associated with an increased number of tetanus booster vaccine prescriptions, the results could be quite different for other educational messages, such as those regarding more expensive or complex vaccines (e.g., HPV vaccine), or those counselling changes in behaviour (e.g., diet and exercise modifications).

Another factor that should be further examined is the benefit of investing in audiovisual devices. In the field of vaccines, proper use of a simple computerized schedule could perhaps achieve similar results, but requires that GPs use it regularly and that patients regularly visit the same doctor. Audiovisual devices must be shown to be cost effective and efficient for purposes other than vaccination.

Several ethical issues should be addressed in the perspective of a widespread use of such audio-visual devices in waiting rooms. Some issues concern the implications of the GPs themselves in terms of conflict between choosing the types of health messages to promote and the potential income they might generate. There is indeed a risk that the choice of audio-visual messages may be guided not by the potential benefits of such messages on the patients' health but by the increase in paid medical procedures that the audio-visual messages may induce. The involvement of the pharmaceutical industry may also be a problem, in the sense that the choice of audio-visual messages may be partly dictated by pharmaceutical sponsorship. Thus, accounting for ethical issues in audio-visual messages will help identifying the foremost priorities in patient's healthcare needs but also assessing the scientific validation of audio-visual messages in terms of their ability to induce health improvements.

Finally, our study did not address a qualitative concept that should be explored: the patients' opinions and experiences. Did the patients enjoy the messages or did they find them annoying? Were they seeking information, or did they feel saturated with information? How well was the health education message received and understood?

## Conclusion

This study shows that an audiovisual message about tetanus booster vaccination broadcast in GP waiting rooms was associated with a significant increase in the number of prescriptions for this vaccination. The effectiveness of audiovisual messages should be explored for other vaccines, including those that are more expensive and difficult to administer. Further studies should also be performed to confirm that this medium can be used to disseminate other types of health information, and that its use is ethical and acceptable to patients.

## Competing interests

The authors declare that they have no competing interests.

## Authors' contributions

CE, FB, JLB, AF prepared the study and designed it in cooperation with DG. CE, SV collected, analyzed and interpreted the data. CE and DG drafted the manuscript. FB, JLB, AF and SV revised and corrected the draft. All authors read and approved the final version of the manuscript.

## Pre-publication history

The pre-publication history for this paper can be accessed here:

http://www.biomedcentral.com/1471-2296/12/104/prepub
